# Colored Leaves of *Epipremnum aureum* Reveal Contrasting Dynamic Photosynthetic and Water-Use Strategies

**DOI:** 10.1021/acsomega.6c04515

**Published:** 2026-07-07

**Authors:** Renan Falcioni, Werner Camargos Antunes, José Alexandre M. Demattê, Marcos Rafael Nanni

**Affiliations:** † Graduate Program in Agronomy, 42487 State University of Maringá, Av. Colombo, 5790, Maringá, Paraná 87020-900, Brazil; ‡ Department of Biology, State University of Maringá, Av. Colombo, 5790, Maringá, Paraná 87020-900, Brazil; § Department of Soil Science, Luiz de Queiroz College of Agriculture, University of São Paulo, Av. Pádua Dias, 11, Piracicaba, São Paulo 13418-260, Brazil

## Abstract

Leaves normally operate under temporally heterogeneous light and experience frequent dark–light transitions as the incident irradiance fluctuates on time scales of seconds to minutes. Under such conditions, the kinetics of photosynthetic induction and stomatal adjustment influence carbon gain, water loss, and intrinsic water-use efficiency (iWUE); however, colored leaves have rarely been examined from this dynamic perspective. We compared three cultivar-associated leaf-color phenotypes of *Epipremnum aureum* (Linden & André) G.S. Bunting (Neon, Golden, and Jade cultivars) during a 150 min dark–light–dark sequence. Time-resolved net CO_2_ assimilation (*A*), stomatal conductance (*g*_s_), transpiration (*E*), iWUE, and chamber leaf temperature were analyzed together with photosynthetic pigments, hyperspectral reflectance, and stomatal and epidermal anatomy. The total chlorophyll and normalized difference vegetation index-type indices increased from Neon to Golden to Jade, whereas the area-based carotenoids and visible reflectance decreased. Steady-state *A* ranged from ≈1.3 μmol CO_2_ m^–2^ s^–1^ in Golden to ≈6.0 μmol CO_2_ m^–2^ s^–1^ in Neon. Neon also showed the fastest photosynthetic induction, the highest steady-state iWUE, the lowest *E* during illumination, and the slowest stomatal closure after darkening. Jade showed intermediate *A* and *g*_s_, the highest illuminated *E*, slower induction, and the fastest closure, whereas Golden had the lowest *A* and *g*_s_, the smallest dynamic ranges, and intermediate *E* despite high abaxial stomatal density and sparse trichomes. These cultivar-associated pigment, optical, anatomical, and physiological traits formed distinct covarying profiles during light transitions. Owing to color–genotype confounding, colored pothos cultivars provide a practical comparative system for studying dynamic photosynthesis and water use under fluctuating light.

## Graphical abstract

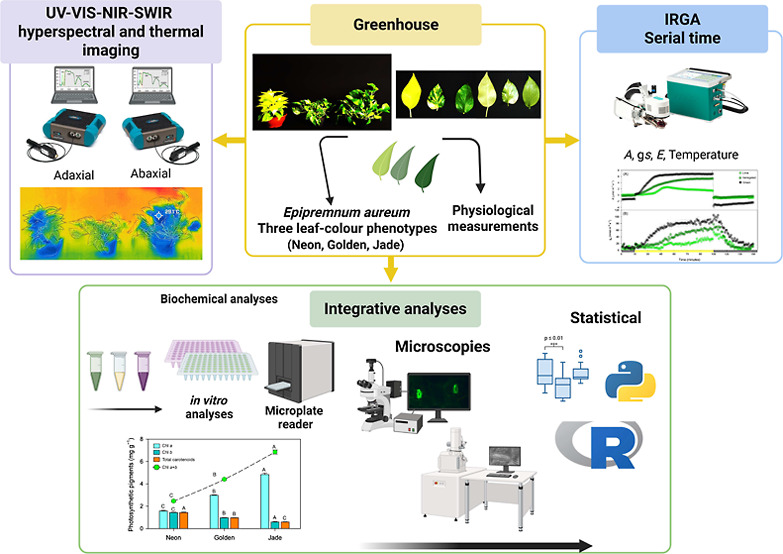

## 1 Introduction

Leaves are the primary organs for light capture and carbon assimilation, yet in many woody evergreen species, they also form conspicuously colored structures.^1–3^ Leaves normally operate under temporally heterogeneous light and experience frequent dark–light transitions as incident irradiance fluctuates on time scales of seconds to minutes throughout the day.^4^ In indoor, greenhouse, or field environments, such dynamics arise when natural or artificial lamps are switched on or off or when sunflecks move across canopies or when plants are moved between locations.^5–7^ Under these conditions, the kinetics of photosynthetic induction and stomatal adjustment strongly influence carbon gain,^8^ water loss, and intrinsic water-use efficiency (iWUE);^9–13^ however, colored leaves have rarely been analyzed from this dynamic perspective. Slow induction of net CO_2_ assimilation (*A*) and stomatal conductance (*g*_s_) following a step increase in light causes substantial penalties for carbon gain, whereas delayed stomatal closure after a decrease in light wastes water without improving assimilation.^14–16^ Understanding how different leaf types modulate these processes and how they balance evaporative cooling with water conservation is, therefore, central to understanding basic plant physiology and plant performance in managed environments with dynamic light.

Colored, chlorophyll-deficient and variegated leaves provide a powerful means to dissect these processes.^2^ Variegation and pale phenotypes arise from altered chlorophyll metabolism and chloroplast development and are typically associated with reduced photosynthetic capacity, modified mesophyll architecture and changes in antioxidant metabolism.^17–20^ These “leaf color mutants” also differ in terms of their stomatal traits and optical properties, which together control within-leaf light propagation, the partitioning of excitation between photochemistry and heat dissipation, and the coupling between *A* and *g*_s_.^2,16^ However, most work on colored leaves has focused on static traits such as pigment contents, steady-state chlorophyll fluorescence, or broad anatomical changes, often in separate experiments.^2,18^ There is still little mechanistic information on how contrasting leaf colors translate into differences in photosynthetic induction, transpiration, leaf temperature, and iWUE during realistic light transitions or on how these physiological patterns are associated with stomatal and epidermal anatomy.

*Epipremnum aureum* (Linden & André) G.S. Bunting is a perennial vine whose leaves span a wide range of colors, from light green to dark green and yellowish or variegated phenotypes with reduced chlorophyll.^21^ It is shade-tolerant and is routinely cultivated in greenhouses and indoor environments, where low and fluctuating irradiance is common.^22–24^ Commercial propagation has produced visually stable clonal cultivars, including Neon (uniform yellow–green), Golden (a mosaic of green and pale yellow tissue), and fully green Jade. These cultivars should not be treated as color classes within one identical genotype: cultivar identity, leaf color, and other genotype-linked traits are inseparable in the present comparison. Developmental and molecular studies in *E. aureum* and other variegated plants have indicated that altered chloroplast development and pigment distribution contribute to pale and mosaic phenotypes.^17,19,21^ It remains unclear whether this visual gradient is accompanied by distinct cultivar-associated combinations of pigment content, optical properties, stomatal and epidermal structure, and dynamic photosynthetic behavior.

Here, we use the Neon, Golden, and Jade *E. aureum* cultivars as a practical comparative system to examine how plants with contrasting foliage phenotypes gain carbon and lose water during time changes in light. The system is experimentally convenient because the plants are readily maintained vegetatively, the cultivars retain stable and readily distinguishable foliage phenotypes, the broad laminae accommodate a 6 cm^2^ gas-exchange chamber and optical probes, and attached leaves permit repeated nondestructive measurements under low-light-relevant conditions.^21–24^ We combine time-resolved gas exchange and chamber leaf-temperature measurements during a dark–light–dark transition with photosynthetic pigments, hyperspectral reflectance and vegetation indices, and quantitative microscopy of stomata, trichomes, and epidermal cells.^25^ The objective is to identify coordinated cultivar-associated patterns rather than to isolate a color-only genetic effect.

We hypothesized that the Neon–Golden–Jade series forms a coordinated gradient in chlorophyll and carotenoid content and in basic hyperspectral indices, reflected in systematic differences in visible and near-infrared reflectance (H_1_). We further hypothesized that this pigment-optical gradient is associated with distinct dynamic responses of *A*, *g*_s_, transpiration (*E*), iWUE, and leaf temperature (*T*_leaf_) during illumination and darkening such that the three cultivars express contrasting carbon–water-use strategies in terms of both magnitude and kinetics (H_2_). Finally, we tested whether the stomatal density, stomatal index, trichome abundance, and epidermal cell packing covaried with the physiological and spectral patterns (H_3_). These hypotheses concern associations within the present comparative design; demonstration of causal mechanisms would require manipulations within a shared genetic background.

## 2 Materials and Methods

### 2.1 Plant Material and Experimental Design

*E. aureum* (Linden & André) G.S. Bunting plants exhibiting contrasting leaf color phenotypes were cultivated in a greenhouse at the Botanic Garden of the State University of Maringá (Maringá, Paraná, Brazil). Vegetative propagules were obtained from commercial stock plants, rooted, and transplanted into 2 L plastic pots filled with a peat-based commercial substrate. The greenhouse was covered with clear plastic film and side curtains and received only natural daylight (no supplementary lighting or blackout). During the growth period, air temperature reflected ambient subtropical conditions typical of Maringá, Paraná State, Brazil (approximately 24–30 °C during the day and 18–22 °C at night), which fall within the recommended range for *E. aureum* cultivation. Relative humidity was maintained between 60% and 80%. The plants were irrigated once daily to drainage with full Hoagland nutrient solution adjusted to pH 5.5, and the pots were allowed to drain freely.

For the physiological assessments, three commercially available leaf-color cultivars of *E. aureum* (Neon, Golden, and Jade) were used. For each cultivar, 10 independent, marketable-sized plants were selected as biological replicates (*n* = 10 per cultivar). On each plant, one healthy, fully expanded, nonsenescent leaf from the midcanopy was selected as the representative leaf. The same attached leaf was prioritized for all nondestructive measurements, and destructive pigment discs and anatomical segments were collected only after those measurements. For Golden leaves, the sampled areas included both green and pale/variegated sectors so that the pigment and gas exchange values represented heterogeneous laminae rather than isolated tissue sectors. Repeated time points or instrument readings from a leaf were not treated as independent biological replicates. The experiment followed a completely randomized design with cultivar/leaf-color phenotype (Neon, Golden, and Jade) as the fixed factor. Because these are distinct clonal cultivars, the design does not separate the effect of color from that of other genotype-associated differences.

### 2.2 Hyperspectral and Thermal Optical Leaf Properties

Leaf optical properties were characterized in situ on the attached laminae of each color phenotype previously selected for physiological measurements. Leaf reflectance-factor spectra (*R*, 350–2500 nm) were acquired with a FieldSpec 3 spectroradiometer (Analytical Spectral Devices, ASD Inc., Longmont, CO, USA) coupled to a 10 mm ASD Contact PlantProbe leaf clip. The instrument comprises a 512-element silicon photodiode array for the visible–near-infrared region (350–1000 nm) and two thermoelectrically cooled graded-index InGaAs photodiodes for the short-wavelength region (1000–1800 and 1800–2500 nm). The PlantProbe internal tungsten–halogen source illuminated the adaxial or abaxial leaf surface, and wavelength-dependent calibration was performed against a Spectralon white reference panel (Labsphere Inc., North Sutton, NH, USA) at the beginning of each measurement sequence. Reflectance was recorded at 1 nm spectral sampling from 350 to 2500 nm, and spectra were later used to compute standard pigment- and water-sensitive vegetation indices, including the normalized difference vegetation index (NDVI), green NDVI (GNDVI), structure-insensitive pigment index (SIPI), water band index (WBI), normalized difference infrared index (NDII), carotenoid reflectance indices 1 and 2 (CRI1 and CRI2), and photochemical reflectance index (PRI), according to their original formulations. The plotted quantity was the untransformed relative reflectance factor *R*(λ) = *S*_leaf(λ)_/*S*_white_(λ), where *S*_leaf_ and *S*_white_ are the wavelength-specific detector signals for the leaf and Spectralon reference (calibration equipment), respectively. Dark calibration was performed automatically by the equipment before measurements. Values were reported directly as fractional reflectance [displayed as percentages for reflectance (%)]; no −log transformation or additional scattering correction was applied to the data.

Longwave infrared thermal images were obtained with a FLIR Vue Pro radiometric thermal camera (FLIR Systems, Danderyd, Sweden; spectral band 7.5–13.5 μm) mounted at a fixed distance and perpendicular to the plant canopy. Images were acquired under greenhouse conditions for the plants selected for physiological assessment. False-color temperature maps were generated with the manufacturer’s software, and the mean canopy/leaf-surface temperature was extracted from regions of interest encompassing each leaf-lamina. These greenhouse FLIR measurements constitute a separate data set from the plant temperature recorded inside the LI-6800 chamber; the two temperature data sets were not pooled or treated as equivalent.

### 2.3 Biochemical Analyses

For photosynthetic pigment quantification, leaf discs (≈1 cm^2^) were excised from representative leaves and immediately processed. In Golden leaves, each disc included both green and pale/variegated tissue in proportions present within the sampled lamina; sector-specific pigment contents were not determined. Pigments were extracted using the chloroform–methanol two-phase protocol of Gitelson and Solovchenko.^26^ The samples were ground in microtubes with chloroform/methanol (2:1, v/v) containing CaCO_3_ until complete extraction, after which distilled water corresponding to 20% of the final volume was added to induce phase separation. The tubes were subsequently centrifuged at 15,000 rpm for 5 min.

The lower (chloroform-rich) phase containing total chlorophylls and carotenoids and the upper (methanol–water) phase containing extra-chloroplastidic pigments (including flavonoids and other phenolic compounds) were transferred to quartz 96-well microplates (200 μL well^–1^) and read in a UV–VIS microplate reader (Biochrom Asys UVM-340; Biochrom Ltd., Cambridge, UK). The absorbance of the chlorophyll–carotenoid phase was recorded at 470, 652, and 665 nm. Chlorophyll *a*, chlorophyll *b*, total chlorophyll (Chl *a* + *b*), and total carotenoids (carotenes and xanthophylls) were calculated using standard equations and expressed on an area basis (mg m^–2^) and a mass basis (mg g^–1^ dry mass), with values corrected for the effective optical path length of the plate by the instrument software.

### 2.4 Gas Exchange Measurements

#### 2.4.1 Dark–Light–Dark Induction Protocol

Gas exchange was monitored with an open infrared gas-exchange system (LI-6800, LI-COR Biosciences, Lincoln, NE, USA) equipped with an LI-6800-01 Multiphase Flash fluorometer head. One representative leaf from each of the 10 independent plants per cultivar was measured in a separate run (*n* = 10 leaves from 10 plants per cultivar) using a 6 cm^2^ chamber. For Golden leaves, the enclosed 6 cm^2^ area contained both green and pale/variegated sectors and therefore provided a whole-area average. The fluorescence function was not activated in this experiment. The head was used as the gas-exchange chamber and integrated with a red/blue LED light source. The reference CO_2_ concentration was set to 400 μmol mol^–1^, the flow was 700 μmol s^–1^, the fan speed was 10,000 rpm, the relative humidity was approximately 60%, and the block temperature was 25 °C. These set points corresponded to a nominal chamber-air vapor pressure deficit of approximately 1.27 kPa. The LI-6800-recorded leaf-to-air vapor pressure deficit (VPD_LEAF_), shown as a time series, ranged from approximately 1.14 to 1.51 kPa across the dark–light–dark sequence. Illumination was supplied by the integrated LI-6800-01 actinic LED array at a 90:10 red/blue photon ratio. The manufacturer-specified peak wavelengths are 625 nm for the red LEDs and 475 nm for the blue LEDs, corresponding nominally to 900 and 100 μmol photons m^–2^ s^–1^, respectively, at the 1000 μmol photons m^–2^ s^–1^ set point. This standardized red-dominant spectrum was selected to drive photosynthetic induction while retaining a blue component that supports stomatal opening; spectral quality was not an experimental factor.

Each run consisted of a 150 min dark–light–dark sequence. After the chamber was closed in darkness (photosynthetic photon flux density, PPFD = 0 μmol photons m^–2^ s^–1^), the leaves were allowed to acclimate for 15 min. The PPFD was then increased to 1000 μmol photons m^–2^ s^–1^ and held constant for 90 min to follow photosynthetic induction. Thereafter, the actinic light was switched off (PPFD = 0 μmol photons m^–2^ s^–1^), and gas exchange was monitored for an additional 45 min to characterize postillumination relaxation. Net CO_2_ assimilation (*A*, μmol CO_2_ m^–2^ s^–1^), stomatal conductance to water vapor (*g*_s_, mmol H_2_O m^–2^ s^–1^), transpiration (*E*, mmol H_2_O m^–2^ s^–1^), and chamber leaf temperature (*T*_leaf_, °C), and leaf-to-air vapor pressure deficit (VPD_LEAF_, kPa) were recorded at 60 s intervals throughout the 0–150 min period.

#### 2.4.2 Derivation of Dynamic Photosynthetic and Water-Use Traits

The LI-6800 time series was exported and processed with custom scripts written in Python (version 3.10). Outliers were removed using a median absolute deviation filter, and each 150 min record was partitioned into dark acclimation (0–15 min), illuminated induction (15–105 min), and postillumination dark relaxation (105–150 min). For each leaf, initial values (*A*_initial_ and *g*_s,initial_) were calculated as the mean over the preillumination dark phase; steady-state values (*A*_steady_ and *g*_s,steady_) were defined as the mean over 95–105 min; final values (*A*_final_ and *g*_s,final_) were defined as the mean over 105–150 min; and maximum values were defined as the highest values reached during illumination.

Changes during opening and closing were defined as Δ*A*_open_ = *A*_steady_ – *A*_initial_, Δ*g*_s,open_ = *g*_s,steady_ – *g*_s,initial_, and Δ*g*_s,close_ = *g*_s,steady_ – *g*_s,final_. First-order exponential models, *y*(*t*) = *y*_f_ + (*y*_0_ – *y*_f_)­e^–*t*/τ^, were fitted separately to the opening (15–105 min) and closing (105–150 min) segments of *A* and *g*_s_. Nonlinear least-squares optimization (SciPy curve fitting) yielded the time constant τ, from which *t*_1/2_ = τ ln(2) and *t*_95_ = – τ ln(0.05) were derived. Kinetic interpretation was based on the observed trajectories and fitted time descriptors. The previously derived normalized carbon loss percentage and the conductance integral labeled as extra water loss were not retained because they could be unstable or misinterpreted as directly measured carbon and water fluxes. For each individual leaf, time was reset to zero at the beginning of each fitted segment; *y*_0_ and *y*_f_ denote the fitted values at the start and asymptote of that segment, respectively, and cultivar means were calculated from the leaf-level parameter estimates. The single-exponential form was used as a parsimonious empirical descriptor for between-cultivar comparison rather than as a mechanistic representation of all biochemical, diffusional, photochemical, and hydraulic processes. Transpiration was interpreted from its directly measured time course rather than fitted independently because *E* is jointly determined by *g*_s_ and VPD_LEAF_, both of which varied during the sequence.

iWUE was calculated at each time point as *A*/*g*_s_. Because *A* is expressed in μmol CO_2_ m^–2^ s^–1^ and *g*_s_ in mmol H_2_O m^–2^ s^–1^, iWUE is reported as μmol CO_2_ mmol^–1^ H_2_O. Initial, steady-state, and final iWUE values were calculated separately for each leaf. Cultivar means and SE were then calculated across the 10 independent leaves from 10 independent plants; the repeated 60 s observations within a leaf were not treated as additional biological replicates. These descriptors were used in the statistical analyses.^9,27^

### 2.5 Microscopic Sample Preparation and Analyses

#### 2.5.1 Sample Preparation

Leaf segments (≈1 mm^2^) were excised from the central region of fully expanded Neon, Golden, and Jade leaves. For quantitative anatomical measurements, a subset of samples was fixed in modified Karnovsky solution (2.5% glutaraldehyde and 2% paraformaldehyde in 0.05 M cacodylate buffer, pH 7.2),^28^ rinsed in buffer, postfixed in 1% osmium tetroxide plus 1.6% potassium ferrocyanide, contrasted overnight in 0.5% uranyl acetate, dehydrated in a graded acetone series, and embedded in Spurr resin. Semithin transverse sections prepared from this embedded material were used for leaf and epidermal thickness determination.

In parallel, fresh freehand transverse sections were cut with a razor blade from nonfixed leaves to visualize mesophyll organization and pigment distribution. For stomatal observations by light microscopy, fresh adaxial and abaxial epidermal peels were gently detached from the lamina, incubated in fluorescein diacetate solution (FDA 0.5%),^29^ and mounted in buffer on microscope slides for immediate analysis. Portions of the fixed dehydrated material were reserved for scanning electron microscopy. All the reagents were of electron microscopy grade and were purchased from Sigma (St. Louis, Missouri, USA) or EMS (Electron Microscopy Sciences, 1560 Industry Road, Hatfield, Pennsylvania, USA).

#### 2.5.2 Optical Microscopy Analyses

Semithin transverse sections (≈1.5 μm) obtained from resin-embedded samples were stained with 1% toluidine blue in borax buffer for 30 s on a 70 °C heating plate and examined with a Leica light microscope equipped with an ICC50 digital camera (Leica Microsystems, Wetzlar, Germany). Digital images were used to determine the total leaf thickness and the thickness of the adaxial and abaxial epidermis in Neon, Golden, and Jade leaves. Measurements were taken on calibrated images using Image-Pro-Plus.

Fresh freehand transverse sections were used to qualitatively confirm the mesophyll arrangement in the three color phenotypes. Fresh adaxial and abaxial epidermal peels stained with fluorescein diacetate were examined under epifluorescence to visualize viable guard cells and stomatal complexes.

#### 2.5.3 Scanning Electron Microscopy Analyses

Dehydrated samples destined for scanning electron microscopy were subjected to critical-point drying in CO_2_ using a CPD-300 apparatus (Bal-Tec AG, Balzers, Liechtenstein), mounted on aluminum stubs, and sputter-coated with gold (50 mA for 150 s) in an MED010 evaporator (Bal-Tec AG). Adaxial and abaxial leaf surfaces were examined under an FEI Quanta 250 scanning electron microscope (FEI Company, Hillsboro, OR, USA) operated at 20 kV. Digital micrographs were acquired with integrated FEI software and subsequently processed for quantitative analysis. SEM was used as the standard approach for detailed visualization of stomata and epidermal cells on the surfaces of leaves of all three color phenotypes.

#### 2.5.4 Quantification of Epidermal Traits and Leaf Thickness

From semithin transverse sections, total leaf thickness and adaxial and abaxial epidermal thickness were quantified on calibrated images. From SEM micrographs, the stomatal density, epidermal cell density, stomatal index, and trichome (or papilla) density were determined separately for the adaxial and abaxial surfaces of Neon, Golden, and Jade leaves. For each surface and trait, counts were made in one distinct calibrated field per leaf at 40× magnification using a 500 × 500 μm counting frame (0.25 mm^2^). Ten fields from 10 leaves originating from 10 independent plants were analyzed per cultivar for each surface and trait (0.25 mm^2^ per leaf). Densities were expressed as the number of structures per unit of leaf area (no. mm^–2^), and the stomatal index was calculated as the proportion of stomata relative to the total number of epidermal cells plus stomata within each field.

### 2.6 Statistical Analyses

Data homogeneity was assessed using Bartlett’s test.^30^ Quantitative variables, including pigment contents, spectral indices, anatomical traits, and summary parameters derived from gas exchange, iWUE, and chamber-temperature time series (Section 2.4.2), were analyzed by one-way analysis of variance (ANOVA), with cultivar/leaf-color phenotype (Neon, Golden, Jade) as the fixed factor. When ANOVA indicated significant effects, the means were separated by Tukey’s multiple-range test at *P* < 0.05. Results are presented as means ± standard errors of the means (SE), calculated across the 10 independent plants per cultivar. For the serial measurements, each time point represents the mean ± SE across the same 10 leaves (one representative leaf per plant); repeated 60 s readings within a leaf were not treated as independent biological replicates. Pearson correlation coefficients were calculated to explore associations among selected physiological, spectral, and anatomical variables, and correlation matrices were visualized as heatmaps when appropriate. Hyperspectral processing, derivation of gas-exchange analysis, and data visualization were carried out with custom Python (version 3.10) routines and R (R Core Team, 2020), whereas additional statistical procedures and graphing were performed with Statistica (version 12.0) and SigmaPlot (version 15.0).

## 3 Results

### 3.1 Dynamic Photosynthetic Responses and Water Use in Plants with Contrasting Leaf-Color Phenotypes

The three *E. aureum* cultivars differed conspicuously in terms of their whole-plant appearance, with Neon plants showing uniformly yellow–green foliage, Golden plants displaying mosaics of pale and green sectors, and Jade plants bearing fully green leaves (Figure 1). The false-color thermal FLIR maps in Figure 1 represent canopy/leaf-surface temperatures measured under greenhouse conditions (approximately 27–33 °C). They provide a spatial context for canopy temperature but are distinct from the controlled single-leaf chamber-temperature time series shown in Figure 2A.

**Figure 1:**
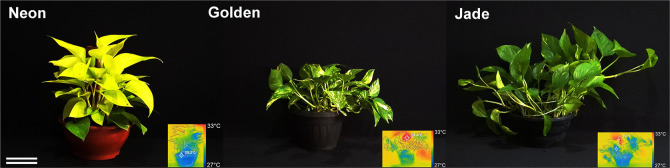
Whole-plant appearance and greenhouse canopy temperature of three leaf-color cultivars of *E. aureum* (Linden & André) G.S. Bunting. Representative Neon (left), Golden (center), and Jade (right) plants were photographed under standardized illumination. Insets show false-color FLIR images acquired under greenhouse conditions; colors represent the leaf surface temperature (approximately 27–33 °C) and are not direct equivalents of the controlled LI-6800 chamber temperature shown in Figure 2A. Scale bar = 15 cm.

**Figure 2:**
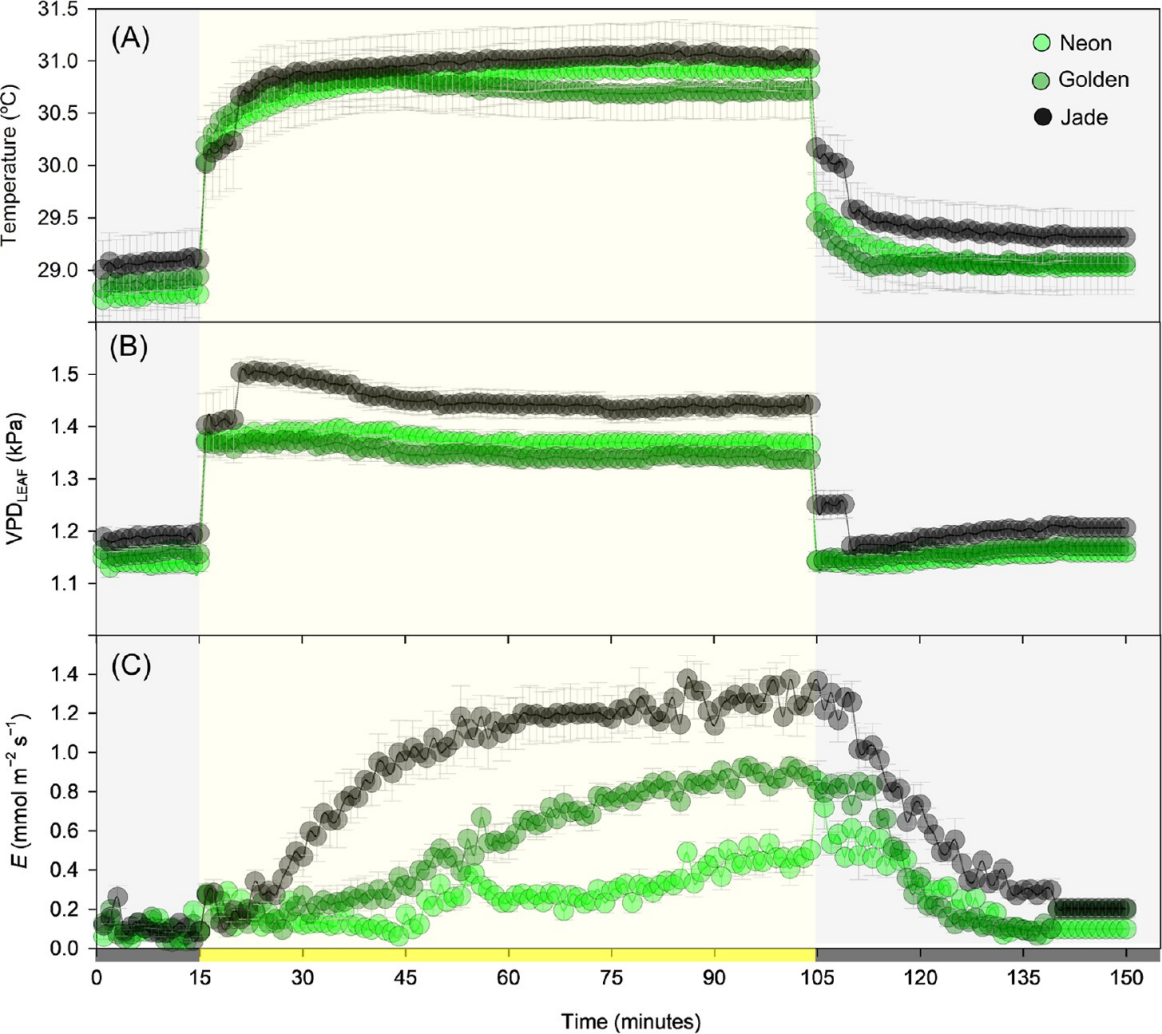
Temporal dynamics of chamber leaf temperature, leaf-to-air vapor pressure deficit, and transpiration in Neon, Golden, and Jade leaves of *E. aureum* during a dark–light–dark transition. (A) Leaf temperature recorded by the LI-6800 under a block temperature of 25 °C and fan speed of 10,000 rpm. (B) Leaf-to-air vapor pressure deficit (VPDleaf, kPa). (C) Transpiration rate (*E*). Leaves were equilibrated in darkness for 15 min, exposed to 1000 μmol photons m^–2^ s^–1^ for 90 min, and returned to darkness for 45 min. Shading along the *x*-axis indicates darkness (gray) and illumination (yellow). Symbols identify cultivars as shown in the legend. At each time point, values are means ± SE for 10 independent leaves, each from a different plant (*n* = 10 per cultivar); repeated 60 s readings within a leaf were not treated as independent replicates. The chamber-temperature data set is distinct from the greenhouse FLIR images in Figure 1.

Inside the LI-6800 chamber, where the block temperature was 25 °C and the fan speed was 10,000 rpm, the leaf temperature increased by approximately 1–2 °C after illumination and decreased after darkening (Figure 2A). Jade leaves generally reached the highest chamber temperatures, Golden leaves were intermediate, and Neon leaves were lowest during the illuminated phase. Leaf-to-air VPD increased from approximately 1.14–1.19 kPa during the initial dark phase to approximately 1.34–1.51 kPa during illumination and returned to approximately 1.14–1.25 kPa after darkening (Figure 2B). Jade generally had the highest VPD_LEAF_ during illumination, whereas Neon and Golden were lower and largely overlapping. Transpiration (*E*) increased after illumination in all the cultivars but decreased after the plants returned to darkness (Figure 2C). During illumination, *E* was highest in Jade leaves, intermediate in Golden leaves, and lowest in Neon leaves. Because the chamber imposed strong forced convection, these temperature differences should not be interpreted as a direct free-air measure of evaporative cooling.

Net CO_2_ assimilation (*A*), stomatal conductance (*g*_s_), and iWUE responded strongly to the light transition (Figure 3 and Table 1).

**Figure 3:**
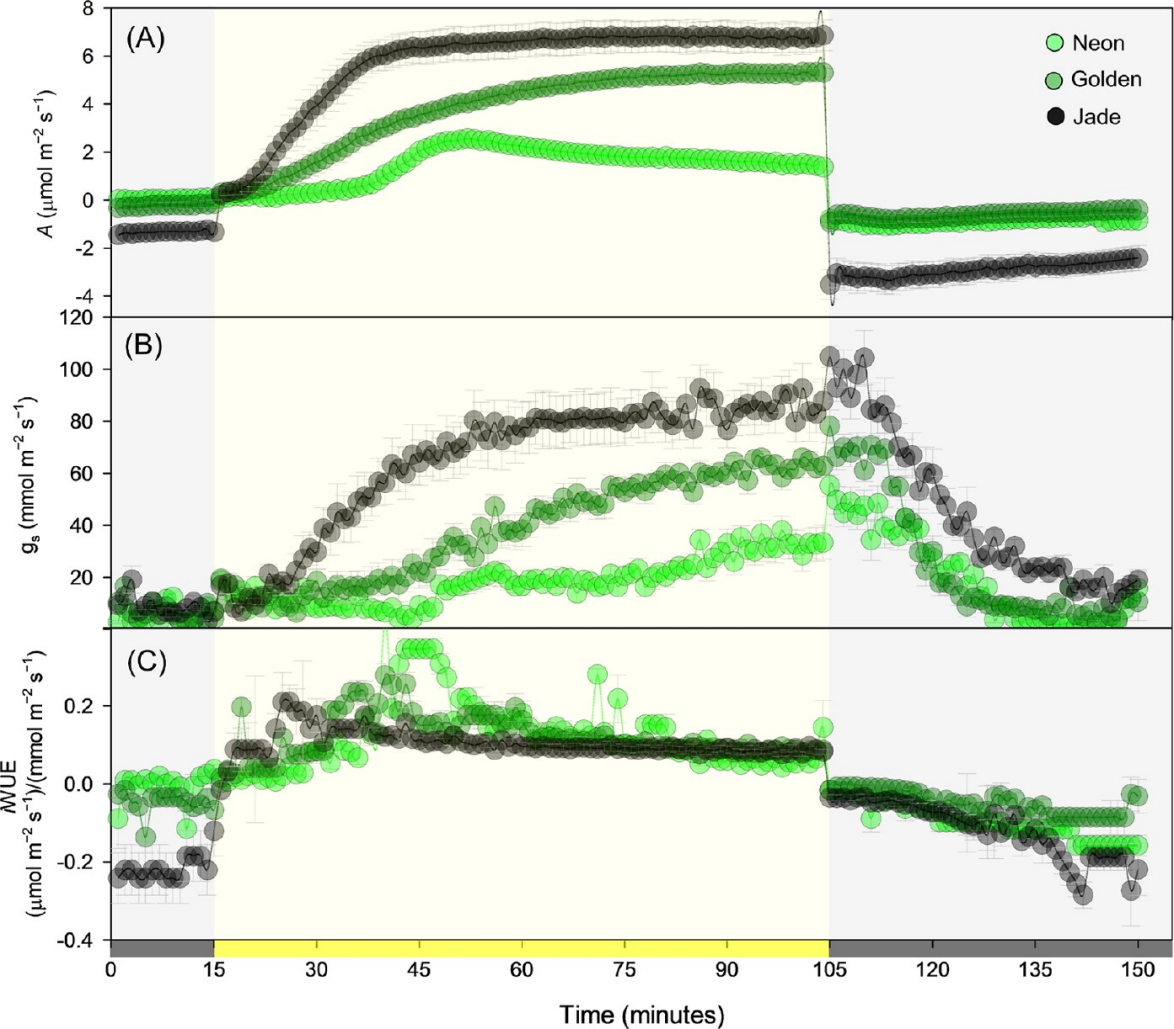
Photosynthetic induction and stomatal responses of Neon, Golden, and Jade leaves of *E. aureum* during a dark–light–dark transition. (A) Net CO_2_ assimilation rate (*A*). (B) Stomatal conductance to water vapor (*g*_s_). (C) Intrinsic water-use efficiency (iWUE = *A*/*g*_s_; μmol CO_2_ mmol^–1^ H_2_O). Measurements followed the regime described for Figure 2, with darkness shown in gray. Black symbols denote Neon, light-green symbols Golden, and medium-green symbols Jade, as shown in the panel legend. Points are the means ± SE for 10 independent leaves from 10 plants per cultivar (*n* = 10 per cultivar); repeated 60 s readings within a leaf were not treated as independent replicates.

**Table 1:** Kinetic Parameters Describing Photosynthetic Induction, Stomatal Opening and Closing, and Intrinsic Water-Use Efficiency (iWUE) in Neon, Golden, and Jade Leaves of *E. aureum* ^a^

parameter group	parameter (units)	Neon leaf	Golden leaf	Jade leaf
parameters of photosynthetic induction (*A*)	*A*_initial_ (μmol m^–2^ s^–1^)	–0.90 ± 0.23 C	0.05 ± 0.03 A	–0.23 ± 0.13 B
	*A*_steady_ (μmol m^–2^ s^–1^)	6.01 ± 0.60 A	1.29 ± 0.17 C	3.08 ± 0.73 B
	Δ*A*_open_ (μmol m^–2^ s^–1^)	7.05 ± 0.70 A	1.24 ± 0.15 C	3.31 ± 0.82 B
	*t*_1/2,open_ (min)	9.18 ± 1.13 B	10.84 ± 0.79 B	17.10 ± 2.07 A
	*t*_95,open_ (min)	39.68 ± 4.87 C	46.85 ± 3.40 B	73.91 ± 8.94 A
stomatal opening and closing kinetics (*g*_s_)	*g*_s,initial_ (mmol m^–2^ s^–1^)	8.78 ± 1.55 A	5.59 ± 0.59 C	7.35 ± 0.57 B
	*g*_s,steady_ (mmol m^–2^ s^–1^)	88.02 ± 8.10 A	33.00 ± 5.59 C	63.50 ± 3.15 B
	Δ*g*_s,open_ (mmol m^–2^ s^–1^)	79.24 ± 7.45 A	27.41 ± 5.77 C	56.15 ± 3.45 B
	Δ*g*_s,close_ (mmol m^–2^ s^–1^)	44.19 ± 2.11 A	15.16 ± 3.21 C	38.91 ± 2.62 B
	*t*_1/2,open_ (min)	38.64 ± 13.48 C	59.60 ± 16.19 B	70.63 ± 9.42 A
	*t*_1/2,close_ (min)	12.80 ± 1.01 A	10.10 ± 0.46 B	8.39 ± 0.59 C
leaf-type effects on intrinsic water-use efficiency (iWUE)	iWUE_initial_ (μmol CO_2_ mmol^–1^ H_2_O)	–0.13 ± 0.05 C	0.01 ± 0.01 A	–0.03 ± 0.02 B
	iWUE_steady_ (μmol CO_2_ mmol^–1^ H_2_O)	0.07 ± 0.01 A	0.05 ± 0.01 B	0.05 ± 0.01 B
	iWU*E*_max_ (μmol CO_2_ mmol^–1^ H_2_O)	0.06 ± 0.00 A	0.06 ± 0.00 A	0.07 ± 0.00 A
	iWUE_final_ (μmol CO_2_ mmol^–1^ H_2_O)	–0.06 ± 0.01 B	–0.06 ± 0.01 B	–0.02 ± 0.01 A
	ΔiWUE_open_ (μmol CO_2_ mmol^–1^ H_2_O)	0.08 ± 0.01 A	0.07 ± 0.01 A	0.05 ± 0.01 B
	ΔiWUE_close_ (μmol CO_2_ mmol^–1^ H_2_O)	0.20 ± 0.03 B	0.45 ± 0.20 A	0.09 ± 0.02 C

a The photosynthetic parameters included initial and steady-state assimilation, Δ*A* open, and the times to 50% and 95% induction. Stomatal parameters include initial and steady-state *g*_s_, Δ*g*_s_, open, Δ*g*_s_, close, and opening and closing half-times. iWUE descriptors comprise initial, steady-state, and final values and are reported in μmol CO_2_ mmol^–1^ H_2_O. The values are the means ± SE for 10 independent leaves from 10 independent plants per cultivar (*n* = 10). Different capital letters within rows indicate significant differences among the cultivars (*P* < 0.05).

In the dark, *A* was near zero or slightly negative for all the cultivars. After illumination, *A* increased to positive steady-state values that differed among the cultivars: Neon leaves had the highest *A*_steady_, Jade leaves had intermediate values, and Golden leaves had the lowest values (Table 1). Δ*A*_open_ followed the same ranking. The half-time and time to 95% induction were shortest in Neon and longest in Jade, with Golden occupying an intermediate position. Interpretation, therefore, focuses on the directly observed *A* trajectories and fitted induction times rather than on a normalized carbon loss percentage (Table 1 and Figure 3).

Stomatal conductance increased in parallel with *A* but showed greater absolute differences among the cultivars (Figure 3B and Table 1). Neon leaves presented the highest initial and steady-state *g*_s_, Jade leaves presented intermediate values, and Golden leaves presented the lowest values. The amplitude of the stomatal opening followed the same order. Stomatal opening was fastest in Neon and slowest in Jade. After the light was switched off, the *g*_s_ decreased in all of the cultivars; closure was fastest in Jade, slowest in Neon, and intermediate in Golden, as indicated by *t*_1/2,close_. No conductance integral was interpreted as directly measured water loss; postillumination water use is instead discussed using measured *E* and stomatal-closing kinetics.

iWUE showed a marked temporal pattern (Figure 3C and Table 1). Immediately after illumination, the iWUE increased from near-zero or slightly negative dark values to a positive plateau. The steady-state iWUE was highest in Neon and slightly lower in Golden and Jade. After darkening, iWUE decreased and could become negative because respiratory CO_2_ efflux occurred while stomatal conductance remained positive; these dark-phase ratios should not be interpreted as productive water-use efficiency.

### 3.2 Pigment Contents and Optical Properties along the Leaf-Color Gradient

The photosynthetic pigment contents differed strongly among the different leaf-color phenotypes (Figure 4). Expressed on an area basis, total chlorophyll (Chl *a* + *b*) was lowest in Neon, intermediate in Golden, and highest in Jade leaves (Figure 4A,B). Chlorophyll *a* was the dominant pigment in all the phenotypes and showed the same increasing trend, whereas chlorophyll *b* increased less sharply. The total carotenoid content was highest in Neon leaves and lowest in Jade leaves on an area basis, resulting in a progressive increase in the chlorophyll/carotenoids ratio along the Neon–Golden–Jade series (Figure 4A). When expressed on a mass basis, total chlorophyll and chlorophyll *a* were again lowest in Neon leaves, intermediate in Golden leaves, and highest in Jade leaves, whereas carotenoid concentrations varied less strongly among the phenotypes (Figure 4B).

**Figure 4:**
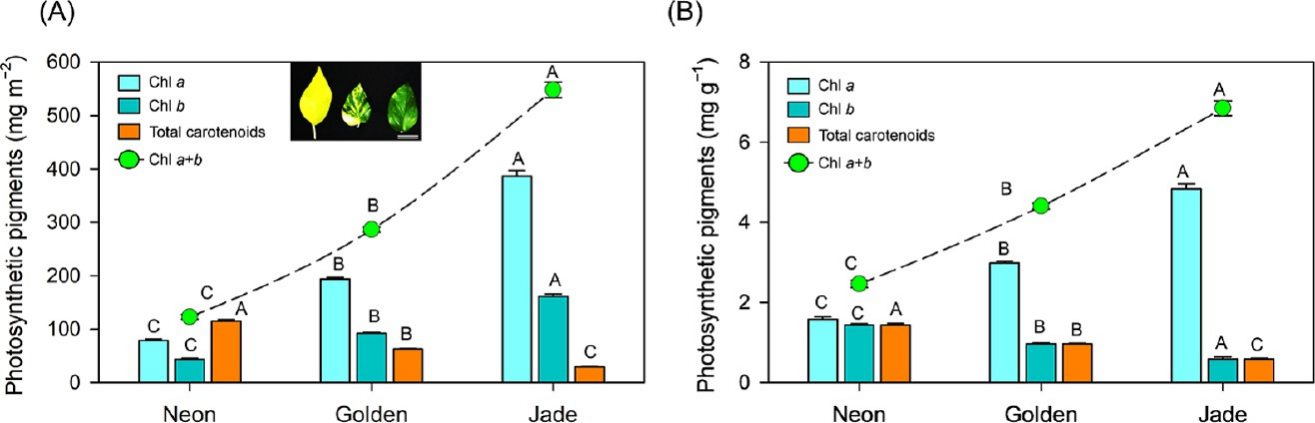
Photosynthetic pigment contents in leaves of three leaf-color phenotypes (Neon, Golden, and Jade) of *E. aureum* (Linden & André) G.S. Bunting. (A) Pigments expressed on an area basis (mg m^–2^). (B) Pigments expressed on a mass basis (mg g^–1^ dry mass). The bars show the levels of chlorophyll *a* (Chl *a*, light cyan), chlorophyll *b* (Chl *b*, dark cyan), and total carotenoids (Car, orange). The dashed line with green circles indicates total chlorophyll (Chl *a* + *b*). The inset in (A) shows representative Neon, Golden, and Jade leaves. Scale bar (bottom right) = 5 cm. The data are presented as the means ± SEs (*n* = 10 independent plants per phenotype). Different capital letters above the bars indicate significant differences among the phenotypes within each pigment class (*P* < 0.05). Each significance letter should be read within a given pigment class across cultivars; adjacent letters above different pigment variables are not intended to form a cultivar-specific code.

These pigment gradients were reflected in the leaf reflectance spectra and vegetation indices (Figure 5). Adaxial and abaxial reflectances in the visible region (500–700 nm) were highest in Neon leaves and lowest in Jade leaves, which is consistent with their lower and higher chlorophyll contents, respectively (Figure 5A,B). In the near-infrared and shortwave infrared regions, the reflectance spectra were more similar among the phenotypes, with only modest shifts in amplitude. The vegetation indices derived from the spectra captured different aspects of these patterns: the NDVI and GNDVI increased from Neon to Golden to Jade, whereas the SIPI decreased along the same series. WBI and NDII differed little and showed no significant cultivar effect, whereas CRI1 and CRI2 were lowest in Neon and highest in Jade. The differences in the PRI were small in magnitude but statistically significant, with that of Jade generally the greatest (Figure 5C,D). Thus, the spectral metrics distinguished the three cultivar-associated leaf types while reflecting different pigment and optical attributes.

**Figure 5:**
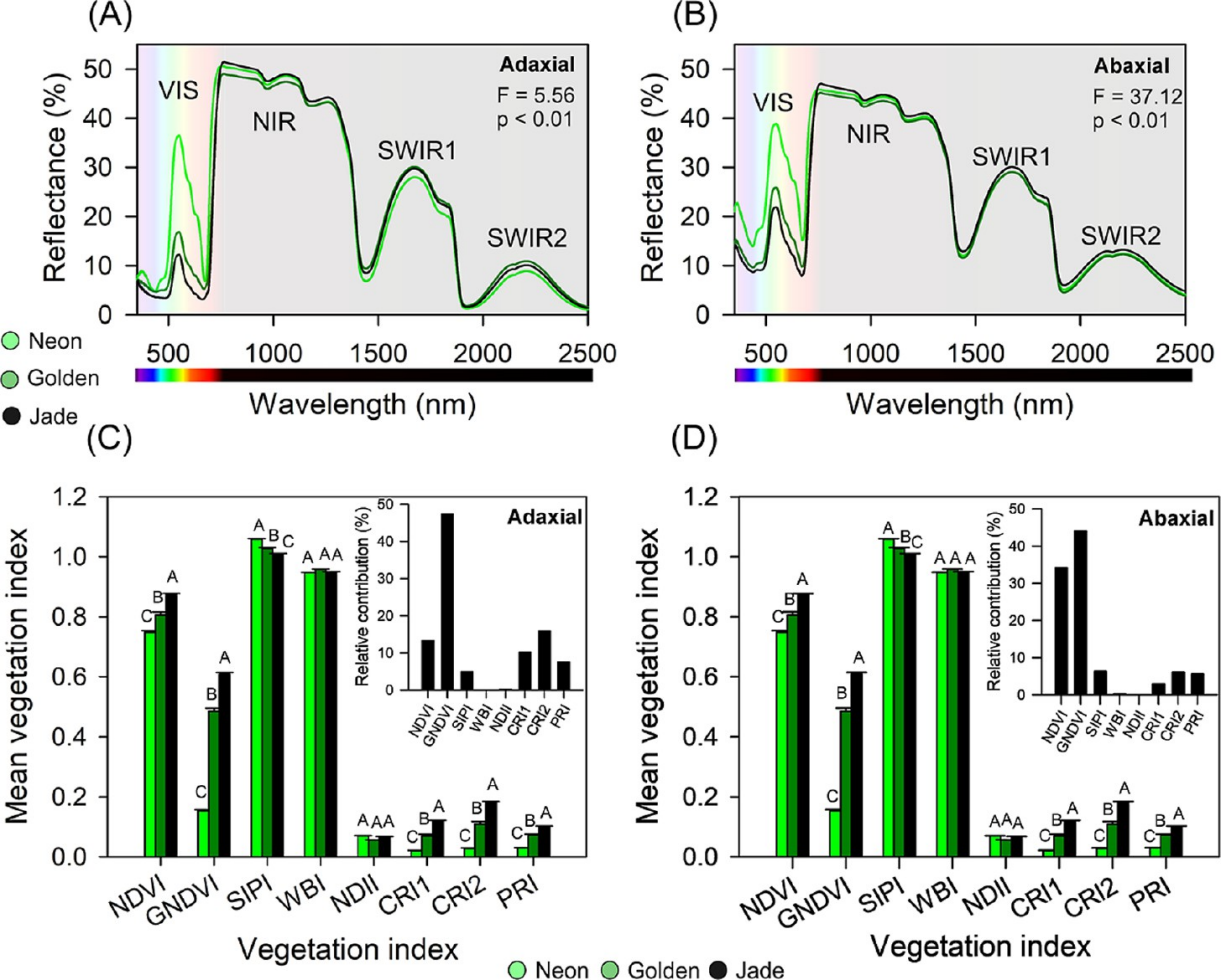
Hyperspectral reflectance and derived vegetation indices for leaves of three leaf-color phenotypes (Neon, Golden, and Jade) of *E. aureum* (Linden & André) G.S. Bunting. (A,B) Mean leaf reflectance spectra (350–2500 nm) for the adaxial (A) and abaxial (B) surfaces. Major spectral regions are indicated: visible (VIS), near-infrared (NIR), and short-wave infrared bands (SWIR1 and SWIR2). Line colors correspond to the phenotypes of Neon (light green), Golden (medium green), and Jade (black). (C,D) Vegetation indices calculated from adaxial (C) and abaxial (D) reflectance: normalized difference vegetation index (NDVI), green normalized difference vegetation index (GNDVI), structure-insensitive pigment index (SIPI), water band index (WBI), normalized difference infrared index (NDII), carotenoid reflectance indices 1 and 2 (CRI1, CRI2), and the photochemical reflectance index (PRI). The bars represent the means ± SEs (*n* = 10 leaves from 10 independent plants per phenotype). Different letters within each index indicate significant differences among the phenotypes (*P* < 0.05). Insets show the relative contribution of each vegetation index to discrimination among leaf types.

### 3.3 Stomatal and Epidermal Anatomy in Different Leaf Types

Microscopic observations revealed similar basic stomatal architecture in all the phenotypes (Figure 6). Light microscopy revealed kidney-shaped guard cells containing multiple chloroplasts and surrounded by irregular epidermal pavement cells on both leaf surfaces (Figure 6A–F). Fluorescein diacetate staining indicated the presence of metabolically active guard cells and adjacent epidermal cells in all the leaf types (Figure 6G–I). Scanning electron micrographs revealed intact stomatal complexes embedded in a moderately sculptured epidermis (Figure 6J–L), together with clear differences in pavement-cell patterning between adaxial and abaxial faces and among phenotypes (Figure 7).

**Figure 6:**
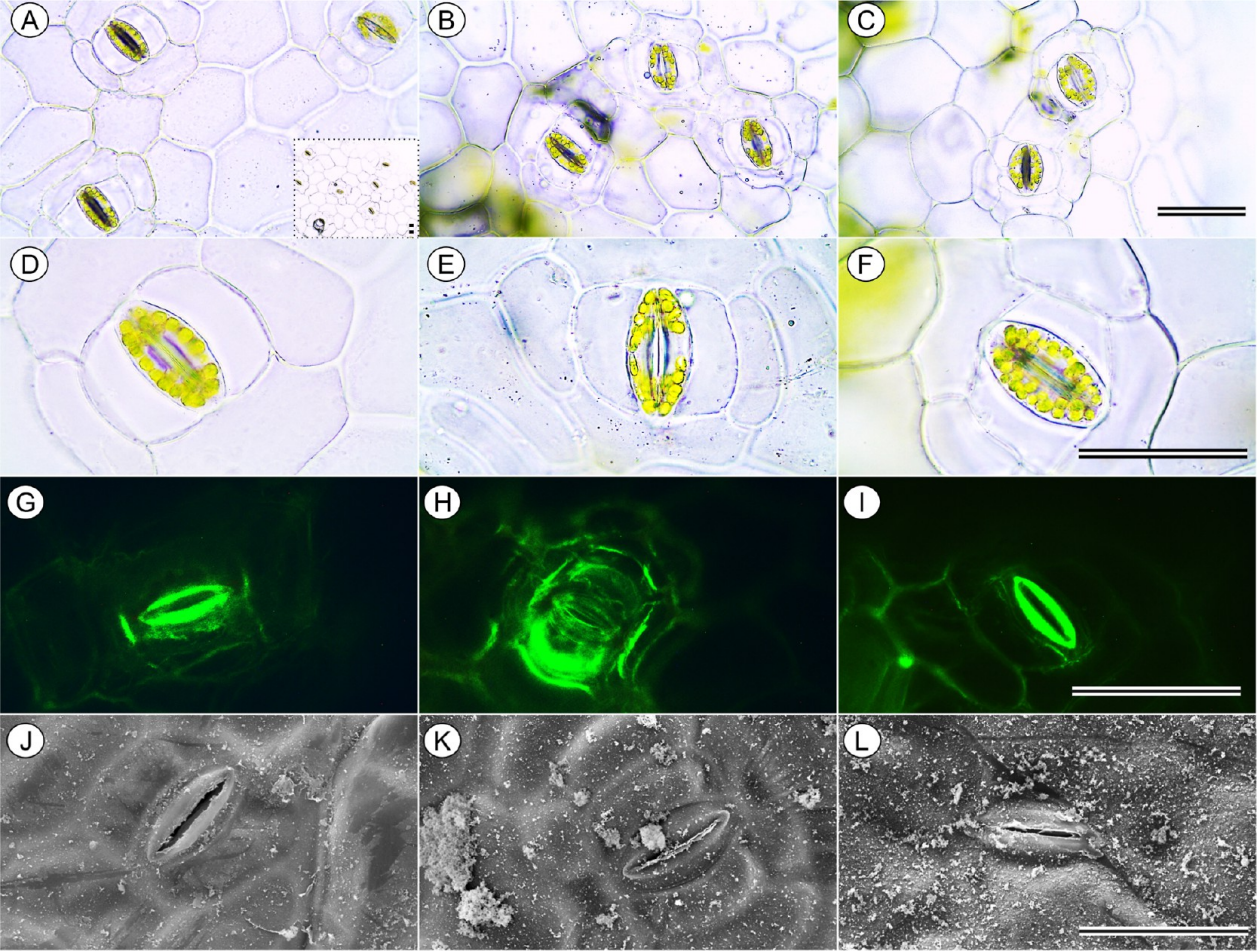
Stomatal structure in leaves of three leaf-color phenotypes (Neon, Golden, and Jade) of *E. aureum* (Linden & André) G.S. Bunting revealed by complementary microscopy techniques. (A–F) Bright-field micrographs of representative stomata surrounded by epidermal cells; guard-cell chloroplasts appear as yellow bodies, indicating multiple chloroplasts per guard cell. (G–I) Fluorescein diacetate (FDA) fluorescence images of stomata, highlighting fluorescein accumulation in guard cells and adjacent epidermal cells. (J–L) Scanning electron micrographs showing external stomatal morphology and the surrounding epidermal surface. Each column corresponds to one leaf-color phenotype (Neon, Golden, and Jade, from left to right). Scale bars = 50 μm.

**Figure 7:**
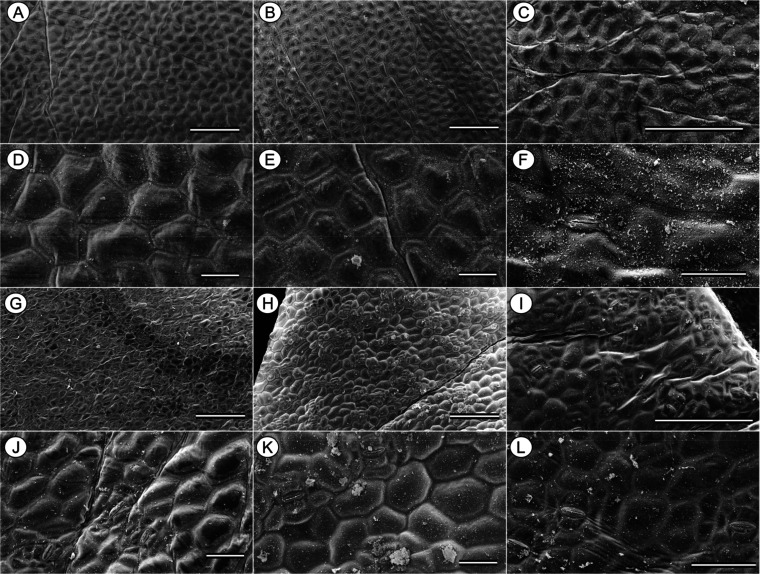
Scanning electron micrographs of the adaxial and abaxial leaf surfaces of three leaf-color phenotypes (Neon, Golden, and Jade) of *E. aureum* (Linden & André) G.S. Bunting. (A–C) Low- magnification views of the adaxial epidermis showing overall epidermal patterning. (D–F) Higher- magnification views of adaxial pavement cells. (G–I) Low- magnification views of the abaxial epidermis. (J–L) Higher-magnification images of abaxial pavement cells. Each column corresponds to one leaf-color phenotype (Neon, Golden, and Jade, from left to right). Scale bars: A–C = 250 μm; D–L = 50 μm.

Quantitative analyses confirmed these anatomical differences (Figure 8). On the adaxial surface, the stomatal density and epidermal cell density did not significantly differ among Neon, Golden, and Jade leaves (Figure 8A,C). The trichome density on the adaxial surface was low for all of the phenotypes (Figure 8B). However, the adaxial stomatal index was lower in Jade leaves than in Neon and Golden leaves (Figure 8D).

**Figure 8:**
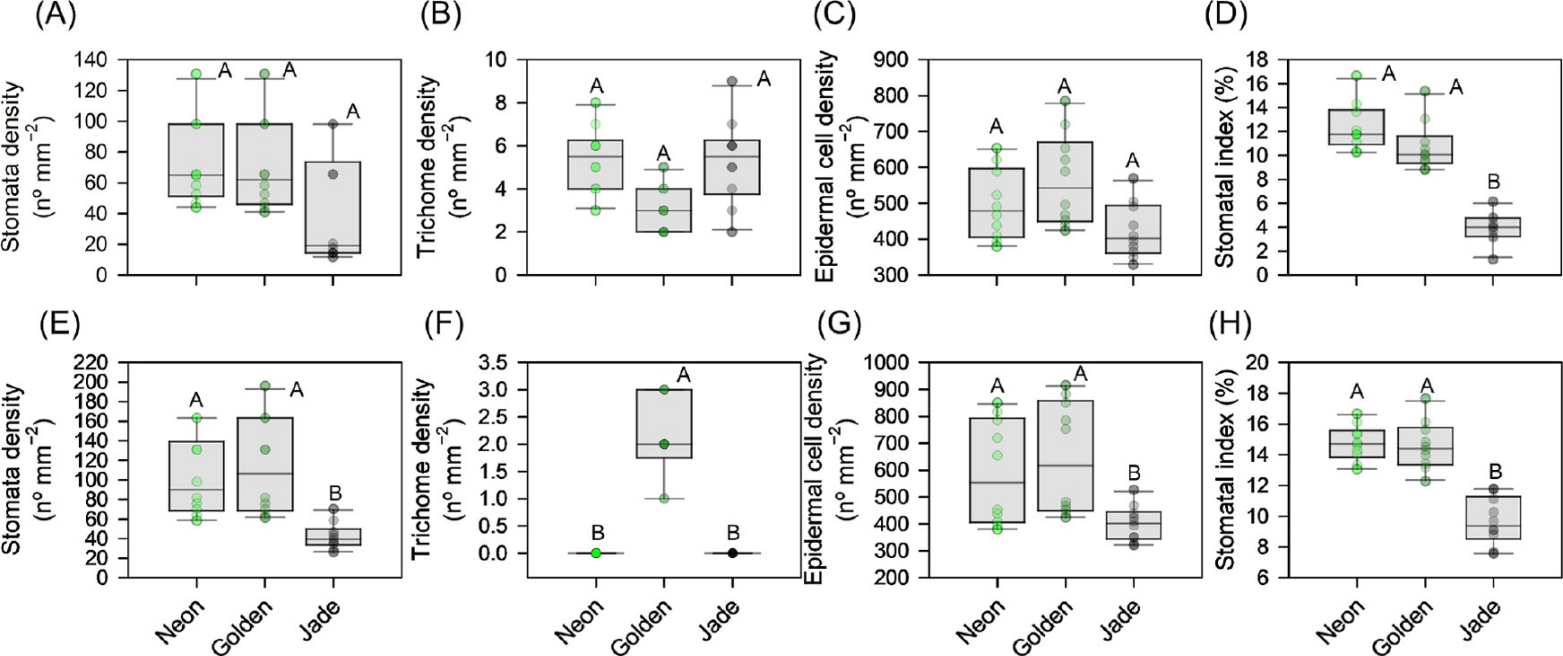
Quantitative stomatal and epidermal traits on the adaxial and abaxial leaf surfaces of three leaf-color phenotypes (Neon, Golden, and Jade) of *E. aureum* (Linden & André) G.S. Bunting. (A–D) Adaxial surface: stomatal density (A), trichome density (B), epidermal cell density (C) and stomatal index (D). (E–H) Abaxial surface: stomatal density (E), trichome density (F), epidermal cell density (G), and stomatal index (H). Boxplots show the median (central line), interquartile range (box), range (whiskers), and individual observations (points) for each phenotype (*n* = 10 leaves from 10 independent plants per phenotype). Different letters denote significant differences among phenotypes within each trait and surface (*P* < 0.05).

On the abaxial surface, where stomata were more abundant, Golden leaves presented the highest stomatal densities, Neon leaves presented intermediate stomatal densities, and Jade leaves presented significantly lower stomatal densities (Figure 8E). Epidermal cell density was also lower in Jade leaves than in Neon and Golden leaves (Figure 8G). Trichomes were virtually absent from the abaxial surfaces of Neon and Jade leaves but occurred at a low density in Golden leaves (Figure 8F). Consistent with these patterns, the abaxial stomatal index was greater in Neon and Golden leaves than in Jade leaves (Figure 8H). Together, these measurements reveal that the leaf-color phenotypes differ in terms of stomatal abundance, the stomatal index, and trichome occurrence, particularly on the abaxial surface.

### 3.4 Alignment between Spectral, Pigment, and Physiological Traits

When considered together, the data sets describe three cultivar-associated trait profiles (Figure 9). Neon combined the lowest chlorophyll contents and NDVI-type indices with high visible reflectance, the highest steady-state *A* and *g*_s_, the highest steady-state iWUE, the lowest illuminated *E*, the fastest photosynthetic induction, and the slowest stomatal closure. Golden displayed intermediate pigment and spectral traits, the lowest *A* and *g*_s_, an intermediate *E* trajectory, the smallest dynamic ranges, high abaxial stomatal density, and sparse abaxial trichomes. Jade combined the highest chlorophyll contents and NDVI-type indices with intermediate *A* and *g*_s_, the highest illuminated *E*, the slowest photosynthetic induction, and the fastest stomatal closure. These are patterns of covariation among cultivars; because cultivar identity and color are confounded, they should not be interpreted as color-only causal effects.

**Figure 9:**
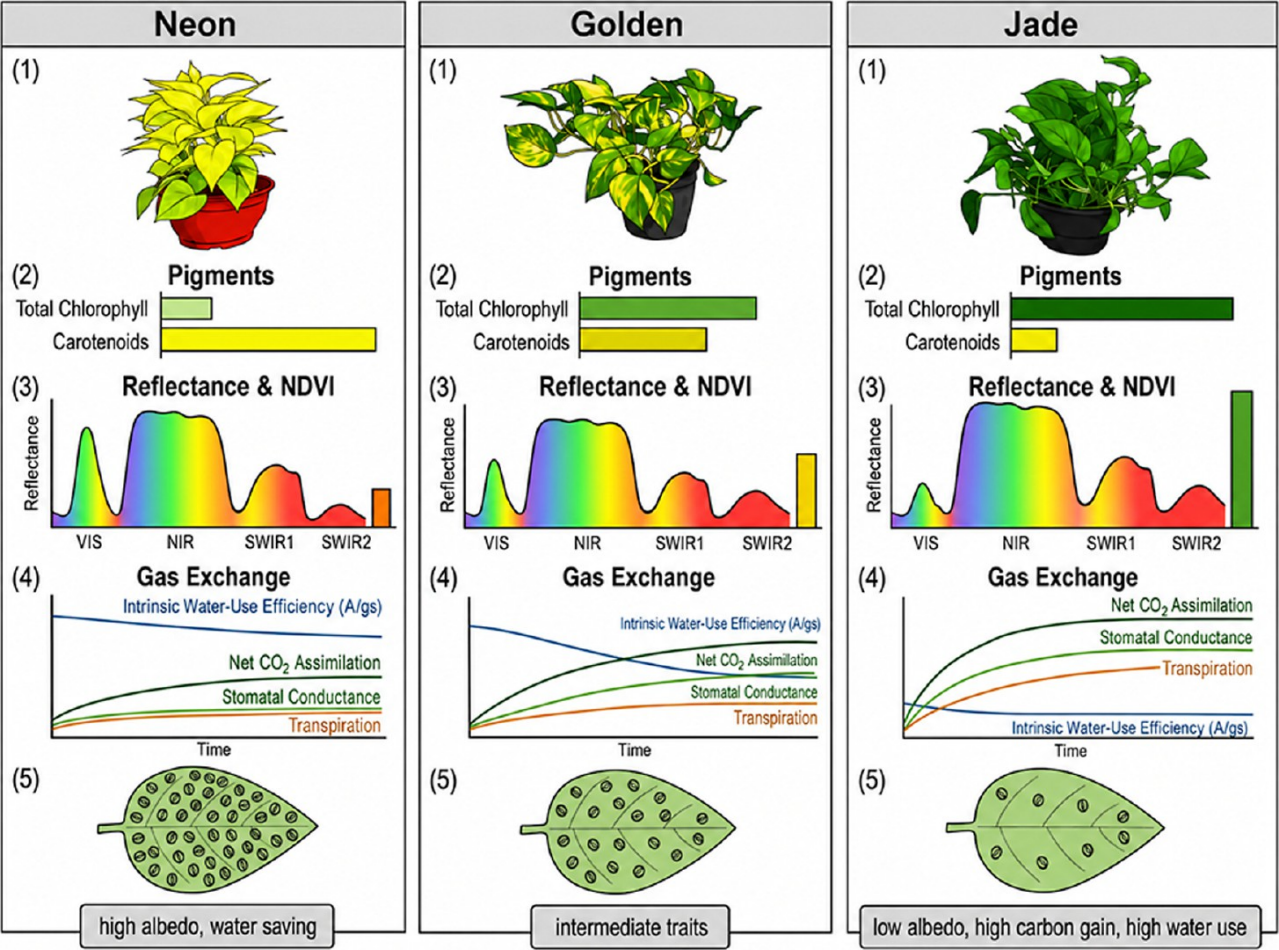
Conceptual synthesis of functional trade-offs among three leaf-color phenotypes (Neon, Golden, and Jade) of *E. aureum* (Linden & André) G.S. Bunting. Panels from left to right summarize the main differences among phenotypes. For each phenotype, (1) a canopy illustration depicts overall leaf color; (2) a bar chart represents photosynthetic pigment composition, highlighting the increase in total chlorophyll and the concomitant decrease in carotenoids from Neon to Jade; (3) a schematic visible reflectance curve and an accompanying NDVI bar indicate high visible reflectance and low NDVI for Neon leaves, intermediate values for Golden leaves, and low visible reflectance but high NDVI for Jade leaves; (4) conceptual gas-exchange trajectories summarize phenotype-specific responses, with net CO_2_ assimilation being highest in Neon, intermediate in Jade, and lowest in Golden, transpiration being highest in Jade, and stomatal conductance being highest in Neon, intermediate in Jade, and lowest in Golden. Intrinsic water-use efficiency (iWUE; *A*/*g*_s_) is highest in Neon and lower in Golden and Jade, where *A* denotes net CO_2_ assimilation and *g*_s_ denotes stomatal conductance; and (5) a cartoon of the abaxial leaf surface indicates relatively high stomatal density in Neon and Golden leaves and lower stomatal density in Jade leaves. Text boxes beneath each panel summarize the emergent strategies, from a high-albedo, low-chlorophyll phenotype with high *g*_s_, high steady-state iWUE, and delayed stomatal closure (Neon), through a mixed-pigment, low-carbon, strongly conservative phenotype (Golden), to a low-albedo, high-chlorophyll, high-carbon, high-water-use phenotype (Jade). The reflectance profiles are conceptual and emphasize the predominant contrasts in the visible region, particularly around the green reflectance peak, as well as reflectance changes in the NIR and SWIR bands. Created with BioRender.com.

## 4 Discussion

### 4.1 Leaf-Color Syndromes of Dynamic Photosynthesis and Water Use

The three *E. aureum* cultivars expressed distinct dynamic photosynthetic behaviors during the dark–light–dark sequence, indicating cultivar-associated carbon–water-use syndromes rather than pigment differences alone.^10,31^ Neon achieved the highest steady-state *A* and *g*_s_ and the fastest *A* induction, Jade reached intermediate *A* and *g*_s_ with slower induction, and Golden operated at low *A* and *g*_s_ with the smallest dynamic range. These contrasts are comparable to the genotypic variation reported for crops and trees, where stomatal and biochemical activation influence carbon gain under fluctuating light.^11,13,32^ Because the present cultivars are distinct clonal genotypes, however, the differences cannot be attributed to leaf color alone.

Neon combined rapid photosynthetic induction with relatively slow stomatal closure. This pattern is consistent with a fast-response strategy in which carbon uptake increases quickly after illumination, but conductance remains elevated longer after darkening.^11^ Jade differed in having the highest measured *E* during illumination but the fastest stomatal closure after light was removed. Golden showed the smallest changes in *A* and *g*_s_, while its *E* trajectory was intermediate between that of Neon and Jade. These statements refer to directly measured trajectories and fitted kinetics; the study does not infer absolute postillumination water loss from a conductance integral. The cultivar contrasts may reflect several genotype-associated properties, including stomatal regulation, hydraulic supply, and photosynthetic capacity, none of which were independently manipulated here.^10,33^

iWUE further distinguished the profiles. Neon maintained the highest steady-state iWUE despite its high *g*_s_, whereas Jade and Golden converged to slightly lower values. The temporal coordination of *A* and *g*_s_ is, therefore, important for interpreting iWUE.^10,13,31,32^ Possible contributions from mesophyll conductance, Rubisco activation, electron transport, and hydraulic signaling remain hypothetical because these processes were not measured. Accordingly, pigment, optical, and anatomical traits are discussed as correlates that may contribute to the observed responses and not as demonstrated causal mechanisms.

The diversity in dynamic behavior is relevant to plants exposed to repeated light transitions. Cultivars with rapid induction may sustain greater carbon gain when repeated light increases, whereas slow closure can maintain conductance after light decreases.^7,13^ *E. aureum* is a practical comparative system for such work because it is readily propagated and maintained as clonal ramets, has stable and visually distinct foliage phenotypes, tolerates low indoor irradiance, has broad leaves compatible with gas exchange and optical sensors, and permits repeated measurements on the same attached leaf.^21–24^ These features strengthen its experimental tractability, but they do not eliminate genotype-color confounding. The measured pigment and spectral gradients are consistent with those of H_1_, and the distinct gas exchange trajectories are consistent with those of H_2_ at the cultivar level; neither hypothesis establishes a causal sequence.^34,35^

### 4.2 Pigment and Optical Traits Covary with Radiative Properties and Temperature Responses

The pigment gradient of the cultivars clearly increased, with the chlorophyll content increasing and the degree of carotenoid dominance decreasing from Neon to Golden to Jade. This pattern is consistent with the broader literature on chlorophyll-deficient and variegated foliage in which altered chloroplast development and pigment distribution generate pale or mosaic tissues.^2,17–21^ All three cultivars retained appreciable amounts of chlorophyll, and guard cell chloroplasts were visible in each. The present study did not measure chlorophyll fluorescence, thylakoid organization, or grana dynamics; the literature on those processes, therefore, provides only biological context and does not provide direct support for an unmeasured mechanism.

Reflectance spectra and vegetation indices mirrored the pigment gradient, with Neon showing the highest visible reflectance and smallest NDVI-type indices and Jade the lowest visible reflectance and largest indices. Lower visible reflectance can increase the amount of absorbed radiative energy, but leaf temperature is also influenced by transpiration, boundary-layer conductance, emissivity, and the measurement environment. The greenhouse FLIR maps and the LI-6800 chamber time series measured different thermal contexts and should not be directly equated. In particular, the 10,000 rpm chamber fan imposed strong forced convection and constrained interpretation in terms of natural evaporative cooling. The Golden values represent averages across the green and pale sectors; thus, within-leaf optical and thermal heterogeneity was not resolved. Similar pigment-optical interactions have been reported in other chlorophyll-deficient foliage.^15,36^

The correspondence among the pigments, hyperspectral indices, and dynamic gas exchange traits revealed that low-dimensional spectral metrics can be used to discriminate among the three cultivars and track major differences in pigments. Comparable associations between hyperspectral reflectance and physiological traits have been reported in forest and crop systems.^34,35,37–39^ Nevertheless, our analysis is correlative and was not designed to establish predictive accuracy or causal pathways. Validation across independent environments and genotypes is needed before spectral indices can be used to predict dynamic photosynthesis in *E. aureum*.

### 4.3 Stomatal and Epidermal Structure as Part of the Syndrome

Stomatal and epidermal traits differed among the cultivars, particularly on the abaxial surface, but these structural patterns did not translate directly to *g*_s_. Golden had the highest abaxial stomatal and epidermal cell densities and a high stomatal index but the lowest *g*_s_ value. Jade had the lowest abaxial stomatal density and stomatal index but an intermediate *g*_s_, whereas Neon combined intermediate density with the highest *g*_s_. This decoupling is consistent with evidence that stomatal density alone is an incomplete predictor of conductance because stomatal size, aperture, kinetics, cuticular conductance, hydraulic supply, and mesophyll demand are important.^10,33^ The present measurements establish an association and not a causal anatomical explanation.

Sparse abaxial trichomes occurred on Golden leaves and may be associated with its low *g*_s_ profile. Trichomes can alter boundary-layer properties under low winds,^40^ and the trichome-to-stomata ratio has been positively associated with intrinsic and whole-plant water-use efficiency in tomato.^41^ However, the LI-6800 chamber fan greatly reduced boundary-layer resistance, the trichome density was low, and the trichomes were not experimentally manipulated. We, therefore, do not attribute Golden’s low *g*_s_ or intermediate *E* causally to trichomes.

Guard-cell chloroplasts were present across all of the cultivars, and fluorescein diacetate staining indicated viable guard cells and epidermal tissues. Guard-cell metabolism can influence osmolyte production and stomatal movement,^31,42,43^ but the number, photosynthetic activity, and signaling function of guard-cell chloroplasts were not quantified here. The low *g*_s_ of Golden may, therefore, reflect differences in guard-cell sensitivity, hydraulic supply, mesophyll signaling, or other cultivar-linked traits. Studies of variegated and chloroplast-defective plants have shown that pigment phenotypes can accompany broader developmental and signaling changes,^2,17–20^ but whether such pathways differ among the present cultivars remains to be determined.

Overall, the anatomical data are consistent with integrated cultivar-associated trait combinations rather than with simple pigment variants. Neon paired high *g*_s_ with intermediate stomatal density and no detected trichomes; Golden combined high stomatal density and sparse trichomes with low *g*_s_; and Jade showed lower stomatal density, intermediate *g*_s_, and rapid closure. These covariations are compatible with broader evidence that stomata and trichomes can covary developmentally and functionally,^40,44^ but direct tests are needed to determine which traits contribute mechanistically to differences in gas exchange.

### 4.4 Functional Implications for Indoor and Ornamental Systems

Colored pothos cultivars are usually described in terms of aesthetic traits, growth habits, and shade tolerance. Our results show that the three commercial cultivars also differ in terms of dynamic photosynthesis and water use. Jade combined the highest illuminated *E* with rapid stomatal closure; Neon combined rapid photosynthetic induction and high steady-state iWUE with slower closure; and Golden maintained the lowest *A* and *g*_s_, the smallest dynamic ranges, and intermediate *E*. These cultivar-associated profiles illustrate different positions along carbon–water trade-off axes.^10,31^ They may be relevant in shaded understories, greenhouses, and indoor cultivation, where irradiance can change abruptly and supplemental lighting is increasingly dynamic.^6,7,45^ The principal evidence is the measured *A*, *g*_s_, *E*, iWUE, and temperature time series together with fitted induction and closure times, not an inferred integral of carbon or water loss.^11,13^

These differences may inform the selection of cultivars for settings that value foliage appearance, resource use, or microclimate effects, although whole-plant growth and long-term water consumption were not measured. The integration of hyperspectral and thermal measurements suggests a route for nondestructive monitoring of potted collections or living walls, but calibration across environments is needed before operational use.^31,34,38,46,51^ Accordingly, the present work provides a comparative physiological basis rather than a mechanistic prescription for cultivar choice.

### 4.5 Limitations and Future Directions

The principal limitation is the confounding effect of color on cultivar/genotype. Neon, Golden, and Jade are commercially distinct clonal cultivars, and the 10 plants per cultivar are clonal ramets; therefore, these differences cannot be attributed uniquely to leaf color or pigment concentration. This limitation is stronger than an assumption of a merely “similar genetic background” and should be considered when every between-cultivar comparison is interpreted. In addition, Golden leaves are mosaics. Pigment discs and the 6 cm^2^ gas-exchange chamber intentionally included both green and pale sectors, so the reported values are whole-area averages and do not resolve sector-specific physiology.^17–21^

Measurements were conducted under one greenhouse growth environment and one dark–light–dark protocol. Dynamic *A*, *g*_s_, and iWUE depend on recent light history, temperature, humidity, VPD, and acclimation.^7,13,32,47^ The nominal chamber-air VPD was approximately 1.27 kPa, and the LI-6800-recorded leaf-to-air VPD (VPD_LEAF_) varied from approximately 1.14 to 1.51 kPa across the sequence (Figure 2B). Moreover, the greenhouse FLIR images (canopy surface temperatures of approximately 27–33 °C) and the LI-6800 chamber measurements (one leaf under a 25 °C block and 10,000 rpm fan) describe different thermal environments. The forced-convection chamber conditions limit the extrapolation of the temperature response to free-air evaporative cooling.

Although the LI-6800 was fitted with a fluorescence-capable head, no chlorophyll-fluorescence protocol was used, and no *F*_v_/*F*_m_, ΦPSII, NPQ, or ETR data were collected for this experiment. Mesophyll conductance, biochemical activation states, hydraulic conductance, and chloroplast ultrastructure were also not measured. Combining gas exchange with fluorescence, mesophyll conductance estimates, and ultrastructural analysis would allow stomatal, biochemical, and photochemical limitations to be separated directly.^4,13,48–50^ Full radiative-transfer modeling and machine-learning analysis could also test whether additional spectral features predict dynamic physiology across independent data sets.^34,38,46,52^ Protocols explicitly designed to resolve multiple induction phases could also compare single- and multiprocess kinetic formulations using information criteria.

The broad principle that chlorophyll deficiency or variegation can coincide with altered optical, anatomical, and physiological traits may extend to other colored-foliage species.^2,18–20,36^ However, the specific ranking observed here—Neon with high *A* and *g*_s_, Golden with the lowest *A* and *g*_s_ but intermediate *E*, and Jade with the highest illuminated *E* and fast closure—should not be generalized without testing other species, cultivars, environments, and within-genotype color sectors. Within these limits, *E. aureum* remains a practical comparative system for repeated, multimodal studies of dynamic photosynthesis under low and fluctuating irradiance.^21–24^

## 5 Conclusions

Colored leaves of *E. aureum* were associated with distinct dynamic carbon–water-use profiles. Neon had the highest steady-state *A* and *g*_s_, rapid photosynthetic induction, and the highest steady-state iWUE with the slowest stomatal closure after darkening. Jade showed intermediate *A* and *g*_s_, slower *A* induction, the highest absolute *E* during illumination, and the fastest stomatal closure. Golden operated at the lowest *A* and *g*_s_, had the smallest dynamic ranges, and had an intermediate *E*.

Pigment composition, visible reflectance, hyperspectral indices, and epidermal traits covaried with these physiological differences. The chlorophyll and NDVI indices increased from Neon to Jade, whereas the visible reflectance decreased; Golden had the highest abaxial stomatal density and sparse trichomes, whereas Jade had the lowest stomatal density. These relationships are observational and should not be interpreted as proof that the measured structural or optical traits cause the gas exchange responses.

Because Neon, Golden, and Jade are distinct clonal cultivars, cultivar identity and leaf color cannot be separated in this design, and Golden values integrate green and pale sectors. Owing to these limitations, the stable phenotypes, broad leaves, shade tolerance, and suitability for repeated nondestructive measurements make *E. aureum* a practical comparative system for studying dynamic photosynthesis and water use. The qualitative framework may inform studies of other colored foliage species, but quantitative rankings require independent validation.

## Data Availability

The processed data supporting the findings are contained in the article.
